# How Unnatural Amino Acids in Antimicrobial Peptides Change Interactions with Lipid Model Membranes

**DOI:** 10.1021/acs.jpcb.4c04152

**Published:** 2024-09-27

**Authors:** Saheli Mitra, Mei-Tung Chen, Francisca Stedman, Jedidiah Hernandez, Grace Kumble, Xi Kang, Churan Zhang, Grace Tang, Ian Daugherty, Wanqing Liu, Jeremy Ocloo, Kevin Raphael Klucznik, Alexander Anzhi Li, Frank Heinrich, Berthony Deslouches, Stephanie Tristram-Nagle

**Affiliations:** † Biological Physics Group, Physics Department, Carnegie Mellon University, Pittsburgh, Pennsylvania 15213, United States; ‡ Center for Neutron Research, National Institute of Standards and Technology, Gaithersburg, Maryland 20899, United States; § Department of Environmental and Occupational Health, University of Pittsburgh, Pittsburgh, Pennsylvania 15261, United States

## Abstract

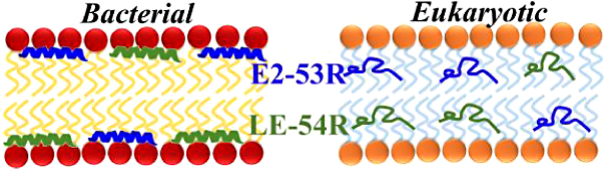

This study investigates the potential of antimicrobial peptides (AMPs) as alternatives to combat antibiotic resistance, with a focus on two AMPs containing unnatural amino acids (UAAs), E2-53R (16 AAs) and LE-54R (14 AAs). In both peptides, valine is replaced by norvaline (Nva), and tryptophan is replaced by 1,2,3,4-tetrahydroisoquinoline-3-carboxylic acid (Tic). Microbiological studies reveal their potent activity against both Gram-negative (G(−)) and Gram-positive (G(+)) bacteria without any toxicity to eukaryotic cells at test concentrations up to 32 μM. Circular dichroism (CD) spectroscopy indicates that these peptides maintain α-helical structures when interacting with G(−) and G(+) lipid model membranes (LMMs), a feature linked to their efficacy. X-ray diffuse scattering (XDS) demonstrates a softening of G(−), G(+) and eukaryotic (Euk33) LMMs and a nonmonotonic decrease in chain order as a potential determinant for bacterial membrane destabilization. Additionally, XDS finds a significant link between both peptides’ interfacial location in G(−) and G(+) LMMs and their efficacy. Neutron reflectometry (NR) confirms the AMP locations determined using XDS. Lack of toxicity in eukaryotic cells may be related to their loss of α-helicity and their hydrocarbon location in Euk33 LMMs. Both AMPs with UAAs offer a novel strategy to wipe out antibiotic-resistant strains while maintaining human cells. These findings are compared with previously published data on E2-35, which consists of the natural amino acids arginine, tryptophan, and valine.

## Introduction

Peptides are attractive candidates for therapeutic applications due to their ability to achieve a high degree of chemical diversity by changes in amino acid primary sequence. Peptide-based drugs have been successfully developed and are widely used in clinical practice.^1,2^ Natural peptides are part of the immune system, with some antimicrobial and immunomodulatory activities.^3−5^ Inspired by naturally occurring antimicrobial peptides (AMPs), scientists have created synthetic versions of AMPs as potential substitutes for conventional antibiotics.^6−8^ Both natural and synthetic AMPs have shown strong and broad-spectrum antibacterial properties in laboratory studies and have been effective in various animal infection models.^9−14^ Therefore, AMPs represent a potential solution to the challenge of treating infections caused by multidrug resistant (MDR) bacteria.

Many AMPs are small in size (12–50 amino acid residues), cationic (a net positive charge of +2 to +13) and amphipathic.^4,15,16^ Several models have been suggested to explain how AMPs work against bacteria, with most emphasizing the disruption of cell membranes. Cationic AMPs are drawn to the negatively charged surface of bacterial cells, which is the basis for their bacterial selectivity.^17^ Despite thorough research, the exact mechanisms by which AMPs interact with membranes and kill bacteria remain unclear for many of these peptides. One hypothesis suggests that when AMPs are added to a suspension of bacterial cells, they undergo “self-promoted uptake” into the cells, crossing the outer membrane or cell wall.^18^ This is followed by disrupting the inner membrane, which ultimately leads to bacterial cell death.^19,20^ AMPs may create pores in the membrane,^21,22^ which can take shapes like “barrel-stave” or “toroidal”.^23−25^ Alternatively, AMPs can act by interfacial activity,^26^ thinning the membrane,^27−29^ segregating lipid domains,^30^ or solvation (referred to as the “carpet” model).^31,32^ Many investigations consider membrane disruption as the primary mechanism of action for AMPs, but other processes like inhibition of cell wall biosynthesis,^33,34^ cell division, and lipopolysaccharide transport^35,36^ may also contribute to their antibacterial effectiveness. These topics have been discussed in recent reviews by Hancock et al.^37,38^

Recent scientific advances have highlighted the potential to enhance the effectiveness and specificity of cationic AMPs. One strategy involves strategically combining specific amino acids. Incorporating positively charged arginine (Arg, R) residues on one face of a helix and hydrophobic valine (Val, V) residues on the other face can improve selectivity. Additionally, extending the peptide chain length and introducing tryptophan (Trp, W) on the hydrophobic face of a helical peptide can enhance antimicrobial activity.^16,39−42^ However, a key challenge in AMP design is balancing antibacterial efficacy with host toxicity.^43^ Some studies caution against using W exclusively in the hydrophobic domain due to its high hydrophobicity and bulky indole ring, which can increase host toxicity.^40^ To mitigate this, we used only three, four or five Ws to the remaining Vs in the hydrophobic domain in our previous studies.^43−45^ Despite these advancements, the transition of AMPs from the lab to the clinic faces obstacles like proteolytic degradation by plasma and bacterial proteases, and hepatic and renal clearance, resulting in loss of antimicrobial activity.^46^ Researchers have explored strategies to enhance AMP stability, including incorporating unnatural amino acids (UAAs), N- and C-terminal modifications, cyclization, multimerizing AMP monomers,^47−54^ and conjugation of AMPs to nanoparticles.^55^ UAAs are not included in the canonical genetic code, but originate from natural or synthetic sources.^56^ Several studies have shown that introducing UAAs enhanced antimicrobial efficacy and proteolytic stability.^57−61^

**Figure 1:**
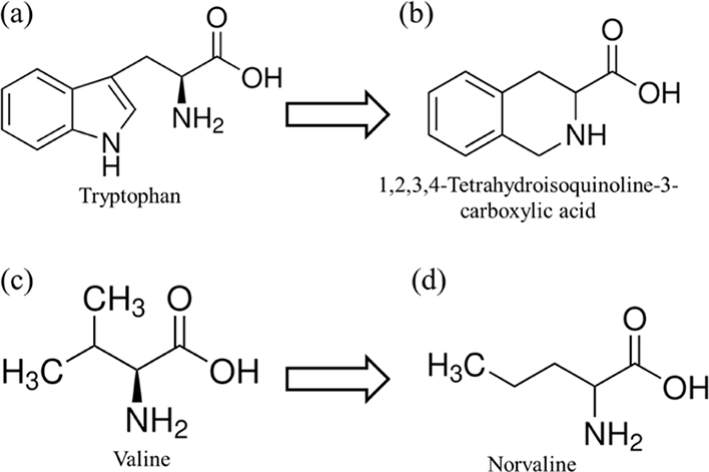
Chemical structures of amino acids. (a) Tryptophan is replaced by (b) 1,2,3,4-tetrahydroisoquinoline-3-carboxylic acid (Tic (X)) and (c) valine is replaced by (d) norvaline (Nva (U)).

In the present study we aim to design peptides with enhanced antibacterial activity and low cytotoxicity. To achieve this, we developed two peptides LE-54R, (14-mer), and E2-53R (16-mer), a derivative of E2-35,^45^ in which Ws and Vs are replaced by 1,2,3,4-tetrahydroisoquinoline-3-carboxylic acid (Tic) and norvaline (Nva), respectively. The chemical structures of the unnatural amino acids are shown in Figure 1. The amino acid sequences and physical attributes of E2-53R and LE-54R are provided in Table 1, where R denotes a peptide containing a UAA. We explored the secondary structures of the AMPs using circular dichroism (CD) measurements to search for a potential correlation with their activity. We used XDS to investigate the effect of these AMPs on the structure of membranes, and to determine their location within different LMMs, and their impact on membrane rigidity and lipid chain order. We used neutron reflectometry (NR) experiments to validate the X-ray results. *In vitro* microbiological assays determined the antibacterial activity and cytotoxicity of E2-53R and LE-54R.

**Table 1:** Amino Acid Sequences of E2-35,^45^ E2-53R and LE-54R and Their Physical Attributes^a^

Peptide	Amino acid sequence	# Residues	Charge	μH	H	μH/H
E2-35	**RR** VW **R**W V**R R**V W**R** WV **RR**	16	+8	0.736	0.363	2.03
E2-53R	**RR** UX **R**X U**R R**U X**R** XU **RR**	16	+8	0.713	0.338	2.11
LE-54R	**RR RR RR R**X XX XU UU	14	+7	0.037	0.378	0.09

a The charged residues are bolded. The hydrophobicity (H) and hydrophobic moment (μH) were determined using the online software HeliQuest (http://heliquest.ipmc.cnrs.fr).

## Experimental Section

### Materials

The synthetic lyophilized lipids 1-palmitoyl-2-oleoyl-*sn*-glycero-3-phosphoethanolamine (POPE), 1-palmitoyl-2-oleoyl-*sn*-glycero-3-phospho-(10-rac-glycerol) sodium salt (POPG), 10,30-bis[1,2-dioleoyl-*sn*-glycero-3-phospho]-*sn*-glycerol sodium salt (TOCL, i.e., cardiolipin), 1-stearoyl-2-oleoyl-*sn*-glycero-3-phosphocholine (SOPC), 1-palmitoyl-2-linoleoyl-*sn*-glycero-3-phosphocholine (PLPC), egg sphingomyelin (ESM), and 1,2-dioleoyl-3-trimeathylammonium-propane chloride salt (DOTAP) were purchased from Avanti Polar Lipids (Alabaster, AL) and used as received. Cholesterol was from Nu-Chek-Prep (Waterville, MN). HPLC-grade organic solvents were purchased from Sigma-Aldrich (St. Louis, MO). In order to create lipid mixtures in molar ratios mimicking bacterial and eukaryotic membranes, lipid stock solutions in chloroform were combined. These molar ratios were used: G(−) inner membrane (IM): POPE/POPG/TOCL (7:2:1 molar ratio), G(+) membrane: POPG/DOTAP/POPE/TOCL (6:1.5:1.5:1),^62^ and eukaryotic membrane, Euk33: SOPC/PLPC/POPE/ESM/cholesterol (15:10:5:3:16.5) (33 mol % cholesterol).^63^ Euk33 for E2-35 had a slightly different composition. Bacterial cation-adjusted Mueller Hinton Broth (MHB2), Test Condition Media, Roswell Park Memorial Institute (RPMI) media, fetal bovine serum (FBS) and phosphate-buffered saline (PBS) were obtained from Millipore Sigma (St Louis, MO). RPMI media contains the reducing agent glutathione and biotin, vitamin B12, and *para*-aminobenzoic acid. In addition, RPMI media includes high concentrations of the vitamins inositol and choline. Because RPMI contains no proteins, lipids, or growth factors, it is commonly supplemented with FBS. FBS contains over 1,000 components such as growth factors, hormones, and transport proteins that contribute to cell growth when supplemented into culture media.^64^ Formaldehyde was obtained from Thermo Fisher (Waltham, MA). The peptides E2-53R (MW: 2979 g/mol) and LE-54R (MW: 2836 g/mol) were purchased in lyophilized form (10 mg in a 1.5 mL vial) from Genscript (Piscataway, NJ) with HPLC/MS spectra corresponding to each designed primary sequence. The traditional antibiotics and colistin were purchased from Millipore Sigma (St. Louis, MO). Amino acid sequences of the peptides and their physical attributes are provided in Table 1.

## Methods

### Antibacterial Assay

The clinical microbiology laboratory of the University of Pittsburgh Medical Center (UPMC) anonymously provided bacterial clinical isolates used for initial screening. Bacteria were stored at −80 °C and retrieved by obtaining single colonies on agar plates before subsequent liquid broth culture. Suspensions of test bacteria were prepared from the log phase of growth by diluting overnight cultures at 1:100 with fresh cation-adjusted MHB2 and incubating for an additional 3–4 h. Bacteria were spun at 3,000*g* for 10 min. The pellet was resuspended in Test Condition Media to determine bacterial turbidity using a Den-1B densitometer (Grant Instruments, Beaver Falls, PA) at 0.5 McFarland units corresponding to 10^8^ CFU/mL.

A standard growth inhibition assay endorsed by the Clinical and Laboratory Standards Institute (CLSI) was modified slightly as previously described.^65^ Bacteria were incubated with each of the indicated peptides in MHB2. The bacterial cells were kept in an incubator for 18 h at 37 °C, linked to a robotic system feeding a plate reader every hour with one of 8 × 96-well plates. The 96-well plates are standard flat-bottom microliter plates purchased from Thermo Fisher (Waltham, MA). This procedure allows the collection of growth kinetic data at A 570 (absorbance at 570 nm) and the examination of growth inhibition in real time (BioTek Instruments, Winooski, VT). Minimum inhibitory concentration (MIC) is defined as the minimum peptide concentration that completely prevents bacterial growth, demonstrated by a flat (horizontal line) growth curve as a function of hourly determinations for 18 h at A570.^43,65^ The assays are typically repeated a second time to ensure accuracy. If the MIC differs from the first assay, a third experimental trial confirms the MIC.

### Determination of Toxicity to Mammalian Cells

Toxicity to eukaryotic cells was examined using human red blood cells (RBCs) and peripheral mononuclear cells (PBMC or white blood cells (WBCs)) as previously described.^43,66^ Briefly, RBCs and WBCs were separated by histopaque differential centrifugation using blood anonymously obtained from the Central Blood Bank (Pittsburgh, PA). For the RBC lysis assay, the isolated RBCs were resuspended in PBS at a concentration of 5%. The peptides were serially diluted 2-fold in 100 μL of PBS before adding 100 μL of 5% RBC to a final dilution of 2.5% RBC to ensure that the A570 of hemoglobin did not saturate the plate reader. In parallel, the RBCs were osmotically burst with water at increasing concentrations to generate a standard curve of RBC lysis. Three technicians independently conducted experiments to ensure reproducibility.

Human WBCs, RPMI and 10% FBS were incubated with each selected peptide for 1 h at 37 °C. The cells were then immediately washed with PBS at 1,000*g* for 7 min, while in a round-bottom 96-well plate. After resuspension in PBS, fixable blue live/dead stain from Life Technologies was added according to the manufacturer’s instructions. The cells were again washed and resuspended in PBS to remove nonspecific stain and then fixed with 4% formaldehyde for 1 h. After washing again with PBS, the samples were stored at 4 °C overnight (in the dark) before examination by flow cytometry using the Novocyte flow cytometer (Agilent Technologies, Santa Clara, CA). Peptide-treated cells were compared with untreated cells for dye incorporation, and data were analyzed using the Novocyte analytical software. Dye incorporation was quantified as percent toxicity directly determined by distinguishing live from dead populations,^66^ which was plotted using GraphPad (Prizm software, San Diego, CA).

### Circular Dichroism (CD)

An extruder (Avanti Polar Lipids, Alabaster, AL) was used to prepare unilamellar vesicles (ULVs) of ∼600 Å diameter. 250 μL of 20 mg/mL multilamellar lipid vesicles were extruded 21 times through a single Nucleopore filter of 500 Å using 0.2 mL Hamilton syringes. The final lipid concentration in the ULVs was 18 mg/mL, as determined gravimetrically. Concentrated ULVs were added to 3 mL of 10 μmol/L (μM) peptide in 15 mM PBS at pH 7 to create lipid/peptide molar ratios between 0:1 and 70:1. Higher molar ratios of lipid-to-peptide were not possible due to absorption flattening in the UV region. We maintained the samples at room temperature for ∼1–4 h before the CD measurement. The samples were loaded into 3 mL quartz cuvettes and placed into the Jasco 1500 CD spectrometer at 37 °C in the Chemistry Department at Carnegie Mellon University. The samples were scanned from 200 to 240 nm 20 times and the results were averaged using the Spectral Analysis software. The temperature was controlled at 37 °C via a Peltier element with water circulation through the sample compartment. Nitrogen gas was introduced at a flow rate between 0.56 and 0.71 m^3^/h to protect the UV bulb. OriginPro 2024 (OriginLab, Northampton, MA) was utilized to perform a Levenberg–Marquardt least-squares fit of the ellipticity traces to four secondary structural motifs representing α-helix, β-sheet, β-turn and random coil.^27,67^ This analysis gives a percentage match of each secondary structural motif to the total sample ellipticity. In order to improve the fit we fixed the ratio of α-helix to β-sheet, and we used specific weighting. Instrument ellipticity was converted to Mean Residue Ellipticity using MRE (deg cm^2^/dmol) = ε × 10^4^/N, where *N* = # amino acids and peptide concentration was always 10 μM.

### Low-Angle X-ray Diffuse Scattering (XDS)

Oriented samples consisting of stacks of approximately ∼1800 bilayers were prepared using the well-established “rock and roll” method, where the substrate is rocked while the lipid in organic solvent rolls over the surface during evaporation.^68^ Mixtures of lipids in chloroform and peptides in trifluoroethanol were combined using a Hamilton repeating dispenser to create lipid:to:peptide molar ratios between 0:1 and 20:1, and excess solvent was evaporated under vacuum. Next, 200 μL of organic solvent (chloroform:methanol (2:1, v/v) or trifluoroethanol:chloroform (1:1, v/v)) was added to the dried film and vortexed; this solution was plated onto a Si wafer (15 mm W × 30 mm L × 1 mm H) inside a fume hood. Basically the sample is rocked during solvent evaporation, where shear force causes an immobile, well-oriented film to form; this was further dried under vacuum for at least two hours. The samples were trimmed to occupy 5 mm W × 30 mm L along the center of the Si substrate. The substrate was fixed to a glass block (10 mm H × 15 mm W × 32 mm L) using heat sink compound (Dow Corning, Freeland, MI). The sample was stored in a refrigerator at 4 °C for several hours. Cold storage before transferring into a well-insulated hydration chamber held at 37 °C caused 100% hydration through the vapor within just 10 min for those samples with a net negative charge. This process is faster than our previous method which requires a Peltier cooler under the sample.^69^ Low-angle XDS (LAXS) data from oriented, fully hydrated samples were obtained at the ID7B2 line at Center for High Energy X-ray Sciences (CHEXS, Ithaca, NY) on two separate trips to the Cornell High Energy Synchrotron Source (CHESS) using X-ray wavelengths of 0.8855 and 0.8856 Å, sample-to-detector (S)-distances of 401 mm and 400.1 mm, beam size 0.25 mm H and 0.35 mm V, and an Eiger 16 M detector. 30-s exposures were carried out in the fluid phase at 37 °C. The flat silicon wafer was rotated from −1 to 6 degrees during the data collection at CHESS to sample all angles of incidence equally. The background was collected by setting the X-ray angle of incidence to −2.0 degrees, where sample scattering does not contribute to the image. For data analysis, backgrounds were first subtracted to remove extraneous air and mylar scattering and the images were laterally symmetrized to increase the signal-to-noise ratio. As the sample nears full hydration, membrane fluctuations occur, producing “lobes” of diffuse X-ray scattering data.^70^ The fluctuations are quantitated by measuring the falloff in lobe intensity in the lateral q_r_ direction. The fitting procedure is a nonlinear least-squares fit that uses the free energy functional from liquid crystal theory,^71^
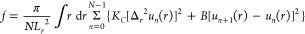
1

where *N* is the number of bilayers in the vertical (*Z*) direction, *L*_*r*_ is the domain size in the horizontal (*r*) direction, and *K*_*C*_ is the bending modulus. *K*_*C*_ describes the bending of an average, single bilayer where *u*_*n*_ is the vertical membrane displacement and B is the compressibility modulus. A higher *K*_*C*_ indicates a stiffer membrane, and a lower *K*_*C*_ indicates a softer membrane. The computer software that implements this fit, NFIT, is freely available by contacting the corresponding author.

### Wide-Angle XDS

Wide-angle XDS (WAXS) was obtained at CHESS, where the same sample that was hydrated for LAXS is X-rayed with a fixed glancing angle of incidence. A gentle nitrogen stream was introduced into the hydration chamber continuously during WAXS data collection to remove significant water scattering in the wide-angle region. Two 30-s exposures are taken at angles of X-ray incidence α = +0.3° and α = −0.3°, where the negative angle image is then subtracted from the positive angle image. The subtraction procedure removes extraneous scatter due to the mylar chamber windows and shadows. The chain–chain correlation appears as strong diffuse scatter projecting upward circularly from the equator; the falloff in intensity yields information about chain order. To obtain an *S*_xray_ order parameter the intensity is first integrated along its radial trajectory, then fit to wide-angle liquid crystal theory.^72^ The chain scattering model assumes long, thin rods that are locally well aligned along the local director (*n*_L_), with orientation described by the angle β. While acyl chains from lipids in the fluid phase are not long cylinders, the model allows the cylinders to tilt (β) in a Maier–Saupe distribution to approximate chain disorder. From the fit of the intensity data using a Matlab computer program,^73^ we obtain *S*_xray_ using eq 2:
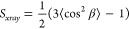
2

We also obtain the RMSE (root-mean-square error), which reports the goodness of the fit.

### Neutron Reflectivity (NR)

NR measurements were performed at the OFFSPEC reflectometer at the ISIS Neutron and Muon Source, Rutherford Appleton Laboratory, Didcot, United Kingdom.^74^ Due to the high contrast between H_2_O and D_2_O, the location of the peptides in the membrane is more easily determined from NR than from X-ray scattering. Reflectivity curves were recorded at 37 °C for momentum transfer values 0.01 Å^–1^ ≤ *q*_*z*_ ≤ 0.33 Å^–1^. The neutron sample cells allowed *in situ* buffer exchange, and a series of measurements on the same bilayer under different isotopic solutions (pure H_2_O and D_2_O) were performed on the same sample. Six mg lipid/peptide mixtures were cosolubilized in chloroform, dried under vacuum and then hydrated for 1–2 h via bath sonication in 1.2 mL 2 M NaCl, creating peptide-containing lipid vesicles. Sparsely tethered lipid bilayer membranes (stBLMs) were prepared on smooth gold-coated (∼140 Å film thickness, 4–9 Å r.m.s surface roughness) silicon wafers by immersing them in a 70:30 mol:mol β-mercaptoethanol:HC18 tether solution in ethanol for at least 60 min, leading to the formation of a self-assembled monolayer (SAM) of both molecules at the gold surface.^75^ SAM-decorated wafers were assembled in the NR cell, and lipid bilayers were completed by fusing vesicles of the desired lipid/peptide mixtures using an osmotic shock procedure.^76^ NR data were sequentially collected after rinsing the NR cell with ∼6 cell volumes of either D_2_O or H_2_O using a syringe. NR data sets collected on stBLMs immersed in isotopically different solutions were analyzed simultaneously (2 data sets per stBLM). One-dimensional structural profiles of the substrate and the lipid bilayer along the interface normal *z* were parametrized with a model that utilizes continuous volume occupancy distributions of the molecular components.^77^ Free-form peptide profiles were modeled using Hermite splines with control points on average 15 Å apart.^78^ The protein extension along the membrane normal determines the number of spline control points and was iteratively refined. A Monte Carlo Markov Chain-based global optimizer was used to determine best-fit parameters and their confidence limits.

## Results and Discussion

### Antibacterial Activity

**Figure 2:**
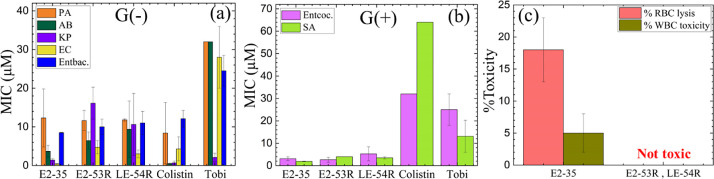
Antibacterial activity and toxicity of E2-35, E2-53R and LE-54R peptides and controls. Selected peptides were examined for MIC against (a) G(−) and (b) G(+) MDR isolates from UPMC. Abbreviations: G(−): *Pseudomonas aeruginosa* (PA), *Acinetobacter baumannii* (AB), *Klebsiella pneumoniae* (KP), *Escherichia coli* (EC), *Enterobacter* (Entbac). G(+): *Enterococcus faecalis* (Entcoc.), *Staphylococcus aureus* (SA) and tobramycin (Tobi). The MICs are the average of strains for each type of bacteria. (c) % Red blood cell (RBC) lysis at 32 μM and % toxicity at 16 μM against freshly isolated human white blood cells (WBCs) were determined by live–dead stain incorporation using flow cytometry. Maximum test concentrations (MTCs) are limited to 16 or 32 μM to ensure each peptide is available for iterative structure–function testing against large panels of antibiotic-resistant clinical isolates. Data are representative of 2–3 experimental trials. Error bars correspond to the standard error of the mean values. Standard deviations are calculated by combining the standard deviations for each bacterial species, σ_Ave_ = √((σ_A_)^2^ + (σ_B_)^2^ + (σ_C_)^2^) /N. E2-35 data were adapted from our previously published paper^45^ with permission.

E2-53R and LE-54R were first tested for antibacterial potency against an MDR panel of G(−) and G(+) bacterial isolates from the University of Pittsburgh Medical Center (UPMC). The Minimum Inhibitory Concentration (MIC) is measured by a horizontal growth curve taken every hour;^43^ these MIC values are plotted in Figure 2a,b. MIC values for the previously reported E2-35 peptide are also included for comparison.^45^ The MICs represent the average values of different strains of each species of bacteria. Remarkably, both R peptides exhibited broad-spectrum activity against both G(−) and G(+) bacterial species, surpassing tobramycin (a conventional antibiotic) with the lowest MIC values. Both peptides demonstrated similar efficacy against G(−) and G(+) bacteria, however overall MICs are lower in G(+) bacteria compared to G(−) bacteria. E2-53R demonstrated inferior antibacterial activity compared to its counterpart E2-35^45^ in G(−) bacteria, however, in G(+) the bactericidal activity of both these peptides were similar.

We used the HeliQuest website (https://heliquest.ipmc.cnrs.fr)^79−81^ to compute hydrophobicity (H) and hydrophobic moment (μH). The physical attributes of both peptides are listed in Table 1. Despite LE-54R having one fewer R and V than E2-53R, it did not notably alter the H values, but the μH value was markedly higher in E2-53R than in LE-54R. Given that both peptides exhibit similar bactericidal activity, the influence of physical attributes on MIC seems negligible in this context. While these calculations are informative, we propose that changes in physical attributes are less crucial than secondary structural alterations of the AMPs and structural modifications in the LMMs.

### Toxicity to Eukaryotic Cells

In order to evaluate the potential harm to eukarytoic cells, we assessed the lytic activity of all peptides in the presence of red blood cells (RBCs) and white blood cells (WBCs). As illustrated in Figure 2c, the data indicate neither of the peptides causes any noticeable toxicity to eukaryotic cells (0%). The presence of cholesterol in the eukaryotic membrane generally reduces the activity of antimicrobial peptides due to either interactions between cholesterol and the peptide or stabilization of the lipid bilayer.^82,83^ van der Waals attractions between the aromatic group in Tic and the four fused-rings of cholesterol may be involved, similar to the cholesterol-binding Crac motif LWYIK that our group studied previously.^84^ This underscores their promising prospects for therapeutic use. This finding contrasts with our previously reported peptide, E2-35, which exhibited some toxicity (<20%).^45^ This is a significant observation as it demonstrates that replacing W and V with the UAAs Tic and Nva, respectively, eliminates toxicity to eukaryotic cells.

### Secondary Structure

**Figure 3:**
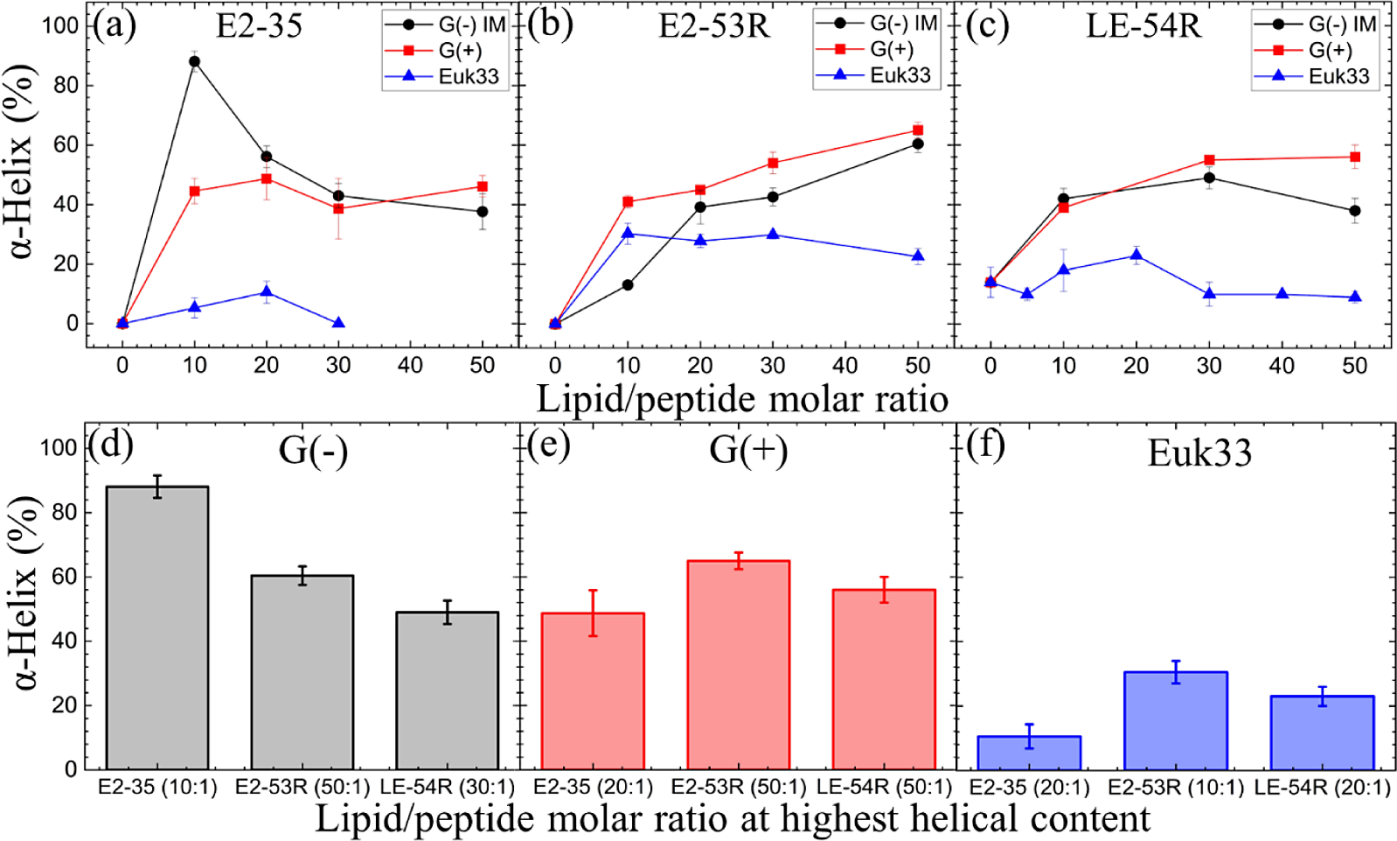
% α-Helix vs lipid/peptide molar ratio of (a) E2-35, (b) E2-53R and (c) LE-54R in G(−) IM, G(+) and Euk33 LMMs. Summary of AMPs’ helical content in three LMMs: (d) G(−) IM (gray), (e) G(+) (red), and (f) Euk33 (blue). The lipid/peptide molar ratio (in parentheses) is for the highest helical content. Standard deviations represent 3–4 fitting results using shape analysis. E2-35 data were adapted from our previously published paper^45^ with permission.

A plot of the % α-helix vs lipid/peptide molar ratio of E2-35, E2-53R and LE-54R in three different lipid membrane models (LMM ULVs) is depicted in Figure 3a–c, while a comparison of the maximum % α-helicity for three peptides is shown in Figure 3d–f. Figures S1 and S2 display the MRE data and % structural motifs for various lipid/peptide molar ratios. Four secondary structural motifs (α-helix, β-sheet, β-turn and random coil) were fitted to the ellipticity data using Levenberg–Marquardt least-squares fitting as described in Materials and Methods. Figure S3 shows examples of three different fits using different weightings for one sample, G(−)/LE-54R(5:1), where the adjusted *R*^2^ improves with specific weighting. Detailed percentage information of four secondary structural motifs in the peptides can be found in Tables S1–S6. Our findings show that E2-53R exhibits a mix of β-sheet and random coil structures in its pure form. On the other hand, LE-54R shows a small degree of helicity, with a higher proportion of β-sheet and random coil structures in its pure form. In contrast, when interacting with G(−) and G(+) LMMs, E2-53R and LE-54R predominantly adopt an α-helical conformation, while in the Euk33 LMM, both primarily adopt a random coil structure. These results highlight the influence of LMM compositions on peptide α-helicity. Interestingly, when compared to previously published CD studies on similar linearly amphipathic peptides (LE-53 and LE-55),^85^ which contained no helicity, the addition of unnatural amino acids promotes the helical structure.

The secondary structures of E2-53R in three LMMs are similar to the results seen with the E2-35 peptide,^45^ indicating that substituting tryptophan (W) and valine (V) with UAAs Tic and Nva does not alter the peptide’s conformation. Recently, Lu et al. studied Pep05 (Lys-Arg-Leu-Phe-Lys-Lys-Leu-Leu-Lys-Tyr-Leu-Arg-Lys-Phe) by substituting L-amino acid residues with d- and unnatural amino acids, such as d-lysine, d-arginine, l-2,4-diaminobutanoic acid (Dab), l-2,3-diaminopropionic acid (Dap), l-homoarginine, 4-aminobutanoic acid (Aib), and l-thienylalanine.^57^ Their CD results suggested that such substitutions did not disrupt the helical structures of the peptides.^57^ Likewise, Oliva et al. reported similar findings with the nona-peptide P9Nal(SS), which contained 2-naphthyl-l-alanine (Nal) and S-tert-butylthio-l-cysteine.^58^

The observed helicity in E2-53R and LE-54R parallels their potent antibacterial activity. E2-53R and LE-54R both exhibit the highest helicity in G(+) LMM and they also kill G(+) bacteria most effectively as shown in Figure 2. This suggests a positive correlation of helicity with the antibacterial efficacy of these peptides. The α-helical structure may facilitate peptides’ interaction with membranes, since hydrophilic and hydrophobic residues line opposite faces of the helix.^86^ Certain amino acids promote helical structure, while others hinder it.^87,88^ Early studies aimed at boosting membrane and antimicrobial activity involved replacing amino acids to increase helicity.^89−91^ For instance, substituting leucine for glycine or deleting glycine at the N-terminus of melittin enhances its helicity and antimicrobial effectiveness.^92^ Conversely, substitutions that prevent melittin from folding into a helix reduce its hemolytic and antimicrobial properties.^93,94^ However, while α-helicity often corresponds to greater efficacy, there are exceptions. For instance, the D8 form of WLBU2, composed of 8 d-enantiomeric valines, exhibited a random coil structure in G(−) LMMs, unlike the predominantly helical structure of WLBU2.^27,95^ Surprisingly, both peptides showed similar efficacy in killing bacteria. Moreover, our recent findings with the linear amphipathic peptide LE-53, which possesses only β-sheet and random coil structures when interacting with bacterial membranes, demonstrated high bactericidal activity.^85^ Since E2-53R and LE-54R have lower helical content in Euk33 membranes, this suggests that cholesterol inhibits helicity. Other investigators have also studied the % helicity of AMPs vs lipid composition with varied results.^96,97^

### Peptide-Membrane Interactions

Structure of peptides can dictate their interactions with membranes^83,98−100^ In addition, both peptides and membranes may alter their structure upon interaction. Experimental studies have explored position, orientation, structure, and effect of peptides on the surrounding lipids.^101−110^ Therefore, a focus on the molecular level interactions between peptides and membranes will lead to a better understanding of biological processes, and help in designing peptides with specific functionalities with potential for therapeutic applications.

#### Membrane Elasticity and Lipid Chain Order Parameter

**Figure 4:**
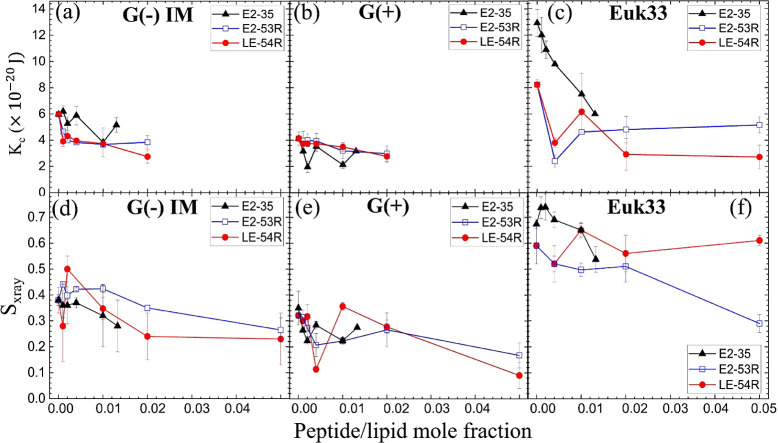
Bending modulus (K_C_) of E2-35 (black triangles), E2-53R (hollow blue squares) and LE-54R (red circles) in (a) G(−) IM, (b) G(+) and (c) Euk33 LMMs. Chain order parameter (S_xray_) of the three peptides in (d) G(−) IM, (e) G(+) and (f) Euk33 LMMs. (colors as in a–c). The standard deviations are from 9 to 18 fittings on the same sample in different positions. E2-35 data were adapted from our previously published paper^45^ with permission.

In the present study, we collected X-ray diffuse scattering in order to understand the change in membrane bending modulus (*K*_*C*_) and lipid chain order parameter (*S*_xray_) of G(−) IM LMM with E2-53R and LE-54R. These results are compared with our previously published data for E2-35.^45^Figure 4a-c shows the elastic bending modulus parameter (*K*_*C*_) of G(−) IM, G(+) and Euk33 LMMs with the three AMPs. A higher value of *K*_*C*_ indicates a stiffer membrane and a lower value indicates a softer membrane. A general softening was observed for E2-53R and LE-54R in G(−) IM and G(+) LMMs, suggesting that similar softening behavior was related to their similar bactericidal activity. By contrast, *K*_*C*_ followed a dramatic nonmonotonic behavior for E2-35 in G(−) IM and G(+) LMMs as shown in Figure 4a,b. We previously suggested that membrane stiffening could result from the interaction of the AMPs with the phosphatidylethanolamine (PE) component of the membranes, whereas membrane softening could result from interaction with the negatively charged lipids, phosphatidylglyercol (PG) and cardiolipin, tested separately.^111^ This could lead to single lipid sequestration into domains with different bending moduli in the bacterial LMMs. At the interface of these domains, defects could arise allowing leakage of ions and water through the domain wall, which would dissipate the bacterial membrane potential. However, the present study suggests that even a monotonic softening could be relevant for bacterial killing. For their interaction with Euk33 LMMs shown in Figure 4c, a general softening was observed for all three AMPs, but since only E2-35 is toxic to eukaryotic cells, we deduce that membrane softening is not the cause of toxicity.

Figure 4d–f plots acyl chain order (*S*_xray_) vs peptide/lipid mole fraction. Higher values of *S*_xray_ signify ordered lipid acyl chains while lower values signify disordered lipid acyl chains. Both AMPs E2-53R and LE-54R caused some degree of nonmonotonicity in lipid chain order which is similar to E2-35 indicating that lipid chain order, unlike *K*_*C*_, may be related to their bacterial killing efficacies through domain formation.

#### Membrane Structural Results

**Figure 5:**
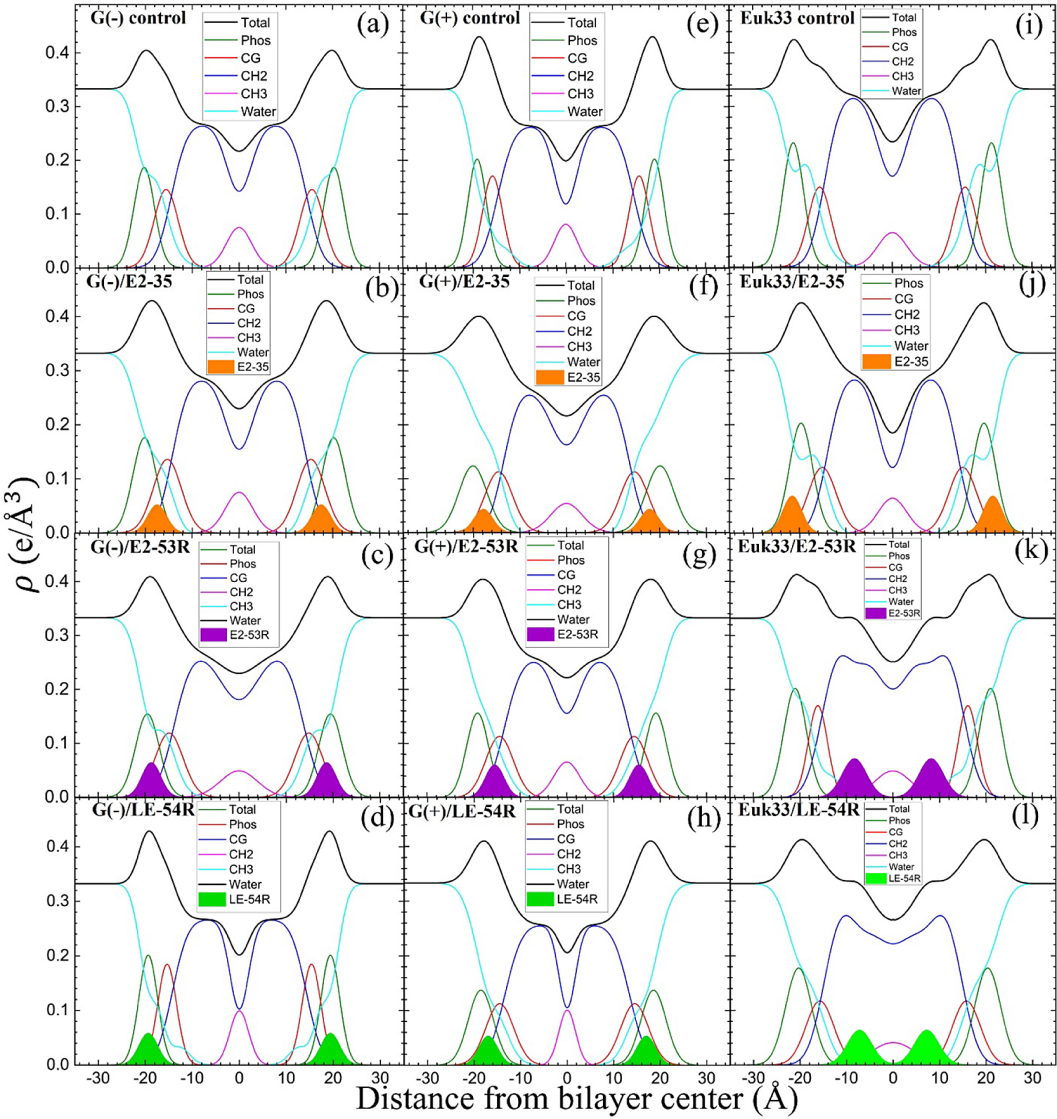
EDPs for G(−) IM LMMs (a–d), G(+) LMMs (e–h) and Euk33 LMMs (i–l) in the presence of E2-35, E2-53R and LE-54R. Component groups in EDPs: phosphate + external headgroup (Phos, green), carbonyl-glycerol (CG, red), CH2 (dark blue), CH3 (magenta), water (cyan), Total (black), E2-35 (filled orange for emphasis), E2-53R (filled purple), LE-54R (filled lime green). The lipid/peptide molar ratio is 50:1. E2-35 data were at 75:1 and adapted from our published paper^45^ with permission.

With the Scattering Density Profile (SDP) program, we can locate the peptides in lipid bilayers to attempt to make a correlation to bacterial killing efficacy. Figure S4 shows form factors |*F*(*q*_*z*_)|, which are the Fourier transforms of the electron density profiles (EDPs) of three LMMs for E2-35, E2-53R and LE-54R shown in Figure 5. SDP considers volumes of lipids, peptides, and their component groups in the bilayer, along with the number of electrons in each component. We fit the form factors by placing a Gaussian envelope for the peptide in three potential locations: the headgroup, hydrocarbon, or a combination of both, then assess the fit quality using chi-square. Generally, the SDP bilayer model fits the XDS form factor data well (Figure S3), resulting in EDPs typical of fully hydrated membranes. The various component groups in EDPs are Phos (phosphate plus outer headgroup), CG (carbonyl/glycerol), CH_2_ (methylene hydrocarbon region containing CH groups), CH_3_ (terminal methyl group), Water (fills volumes around other groups to maintain a total probability of one), and Total (sum of all component groups). Key measures derived from these EDPs include the combined peak-to-peak distance of Phos and CG (*D*_HH_), and the full-width at half-maximal of the hydrocarbon region (2*D*_C_), indicating membrane thickness. The EDP also determines the area per lipid molecule (*A*_L_) using lipid and peptide volumes. A summary of the XDS structural results for the three LMMs used in this study interacting with E2-53R and LE-54R is shown in Table 2.

**Table 2:** Summary of Structural Results from XDS and the Charge/Residue

Sample	Area/lipid *A*_L_ [Å^2^] (±1.0)	*D*_HH_ [Å] (±0.5)	2*D*_C_ [Å] (±0.5)	Net charge/residue
G(−) IM control	71.0	39.8	29.0	-
G(−) IM/E2-35	75.5	38.4	27.3	–0.178
G(−) IM/E2-53R	75.7	38.1	27.3	–0.174
G(−) IM/LE-54R	72.4	38.4	28.5	–0.200
				
G(+) control	72.5	37.2	29.3	-
G(+)/E2-35	79.0	37.5	26.9	–0.209
G(+)/E2-53R	79.3	36.2	26.8	–0.205
G(+)/LE-54R	79.2	36.1	26.8	–0.235
				
Euk33 control	71.5	42.3	29.1	-
Euk33/E2-35	73.6	39.0	28.0	0.006
Euk33/E2-53R	74.8	41.3	30.1	0.010
Euk33/LE-54R	74.9	39.3	29.3	0.010

**Figure 6:**
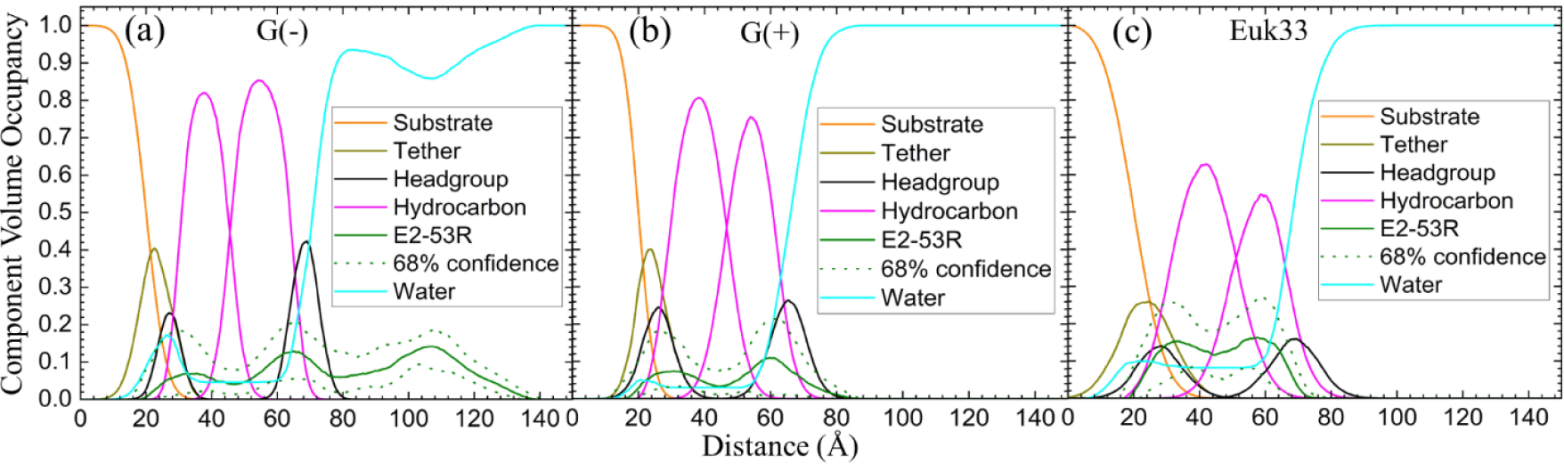
Neutron reflectivity component volume occupancy of E2-53R (a–c) in a single tethered bilayer of G(−) IM, G(+) and Euk33. Component volumes: gold-covered silicon substrate (orange), tether (dark yellow), headgroups (black), hydrocarbons (magenta), E2-53R (olive), water (cyan). The dotted lines represent the 68% confidence limit of the composition-space fit.

XDS data reveal that E2-53R and LE-54R are located in the interfacial region in both G(−) and G(+) LMMs, suggesting that an interfacial location correlates with efficient bacterial killing. This location could be due to their high arginine content, with 8 arginines for E2-53R and 7 arginines for LE-54R. The amino acid arginine contains two extra nitrogens, which allow the guanidinium part of the molecule to form up to six hydrogen bonds.^112^ This unique feature of arginine enables it to interact with phosphate groups in various ways, forming complexes.^113^ Our recent study found that the most effective peptide, E2-35, resides under the CG close to the interfacial region.^45^ When we replaced Trp and Val of E2-35 with the unnatural amino acids Tic and Nva, there was a minor influence on the location of E2-53R in bacterial LMMs as shown in Figure 5c,g. To verify the locations of the peptides, we conducted NR experiments. While E2-53R and LE-54R were studied for XDS, only E2-53R was utilized for NR due to time constraints. NR traces are shown in Figure S5. Figure 6 provides a graphical summary of the membrane location of E2-53R from NR measurements, while Table S7 quantifies the results. When Figure 6 is compared with Figure 5, good agreement between NR and XDS is obtained regarding the peptides’ locations in the membrane.

In our recent studies involving helical amphipathic (E2-35, E2-05) and linear amphipathic (LE-53 and LE-55) peptides, we have observed that peptides E2-35 and E2-05, which are slightly toxic, localize near the headgroup region of the Euk33 bilayer.^45^ Conversely, the nontoxic peptides E2-35K, LE-53, LE-55^45^, LE-54R and E2-53R prefer to be located within the hydrocarbon region of the lipid bilayer. A correlation is thus observed between the peptides’ location within the hydrocarbon region of the bilayer and their lack of toxicity. What is the mechanism of the headgroup location associated with toxicity in both bacterial and eukaryotic membranes? One explanation could be the so-called continuum elastic model (CEM) of Campelo et al. which explains the observation that amphipathic helices induce positive curvature (convex with headgroup on the exterior) through a wedge mechanism, where the helix displaces material near the surface, forcing curvature to relieve the stress.^114^ All-atom simulations predicted an even larger positive curvature when specific chemical effects were included.^115^ A local positive curvature could induce a defect region where water and ions could escape from the cell thus killing it. No change in curvature may occur when a nonhelical, extended peptide lodges in the hydrocarbon interior, which causes only a benign effect.

As indicated in Table 2, all three peptides decrease the thickness of the membrane (measured by 2*D*_C_ and *D*_HH_) in both G(−) and G(+) membranes, regardless of their position within the bilayer. Likewise, the peptides increase the area per lipid (*A*_L_) in both types of membranes. Thus, this suggests that decreases in membrane thickness and increases in area per lipid may be related to the efficient killing of bacteria exhibited by all three AMPs. Even a small, <1 Å, thinning may be enough to contribute to a membrane destabilization.^27^ As for toxicity, 2D_C_ in Table 2 in the Euk33 LMM shows a small thinning only for E2-35 which is toxic, while either no thinning or a thickening caused by the modified peptides which are nontoxic, suggesting that thinning may also be correlated with toxicity.

## Conclusions

This study systematically examines a potential solution to the escalating danger of antibiotic resistance. By investigating the efficacy of AMPs as viable alternatives, the research highlights two specific peptides with unnatural amino acids: E2-53R (16 AAs) and LE-54R (14 AAs). These UAA peptides demonstrate potent activity against both G(−) and G(+) bacteria while ensuring the safety of human cells. Notably, both E2-53R and LE-54R maintain α-helical secondary structures when interacting with G(−) and G(+) LMMs, as revealed by CD spectroscopy, which correlates with their efficacy. Additionally, XDS reveals a monotonic decrease in *K*_*C*_ for both peptides in G(−) and G(+) LMMs, suggesting that membrane softening may also play a role. As for lipid chain order, both E2-53R and LE-54R, as well as E2-35, cause nonmonotonic changes as a function of concentration, suggesting that lipid domain formation may play a role in membrane instability. This research also exposes a crucial link between the interfacial location of these peptides and their effectiveness. As for toxicity, both E2-53R and LE-54R as well as E2-35 lose helicity when interacting with Euk33 LMMs. While E2-35 locates in the headgroup region and is toxic, both E2-53R and LE-54R locate in the hydrocarbon region and are nontoxic, suggesting that AMP location is important in the eukaryotic mimic as well as in the bacterial cell mimic, perhaps through a local curvature mechanism.

## Data Availability

The data underlying this article are available in the article and in its online Supporting Information. Any other details will be shared on reasonable request to the corresponding author.

## Supporting Information Available

Mean residue ellipticity (MRE) results (Figure S1); % structural motifs vs. lipid/peptide molar ratio (Figure S2); % structural motifs (in table) for three different fits of the four secondary structural motifs shown above right from ref (67) to G(−)/LE-54R (5:1) (Figure S3); secondary structural results (% α-helix, β-sheet, β-turn and random coil) for E2-53R and LE-54R peptides in three LMMs (Tables S1–S6) (PDF)
