# Supplementary material for: How Unnatural Amino Acids in Antimicrobial Peptides Change Interactions with Lipid Model Membranes
Source: J Phys Chem B. 2024 Sep 27;128(40):9772–84. doi: 10.1021/acs.jpcb.4c04152 (PMC11472314; doi:10.1021/acs.jpcb.4c04152)
Supplement: Supplementary file 1 — jp4c04152_si_001.pdf [file jp4c04152_si_001.pdf]

How Unnatural Amino Acids in Antimicrobial Peptides Change Interactions with Lipid Model Membranes

Saheli Mitra[1], Mei-Tung Chen[1], Francisca Stedman[1], Jedidiah Hernandez[1], Grace Kumble[1], Xi Kang[1], Churan Zhang[1], Grace Tang[1], Ian Daugherty[1], Wanqing Liu[1], Jeremy Ocloo[1], Kevin Raphael Klucznik[1], Alexander Anzhi Li[1], Frank Heinrich[1,2], Berthony Deslouches[3], Stephanie Tristram-Nagle[1]

[1]Biological Physics Group, Physics Department, Carnegie Mellon University, Pittsburgh, PA 15213, USA; [2]Center for Neutron Research, National Institute of Standards and Technology, Gaithersburg, MD 20899, USA; [3]Department of Environmental and Occupational Health, University of Pittsburgh, Pittsburgh, PA 15261,USA

*Corresponding author's email: stn@cmu.edu , Phone: 412-268-3174

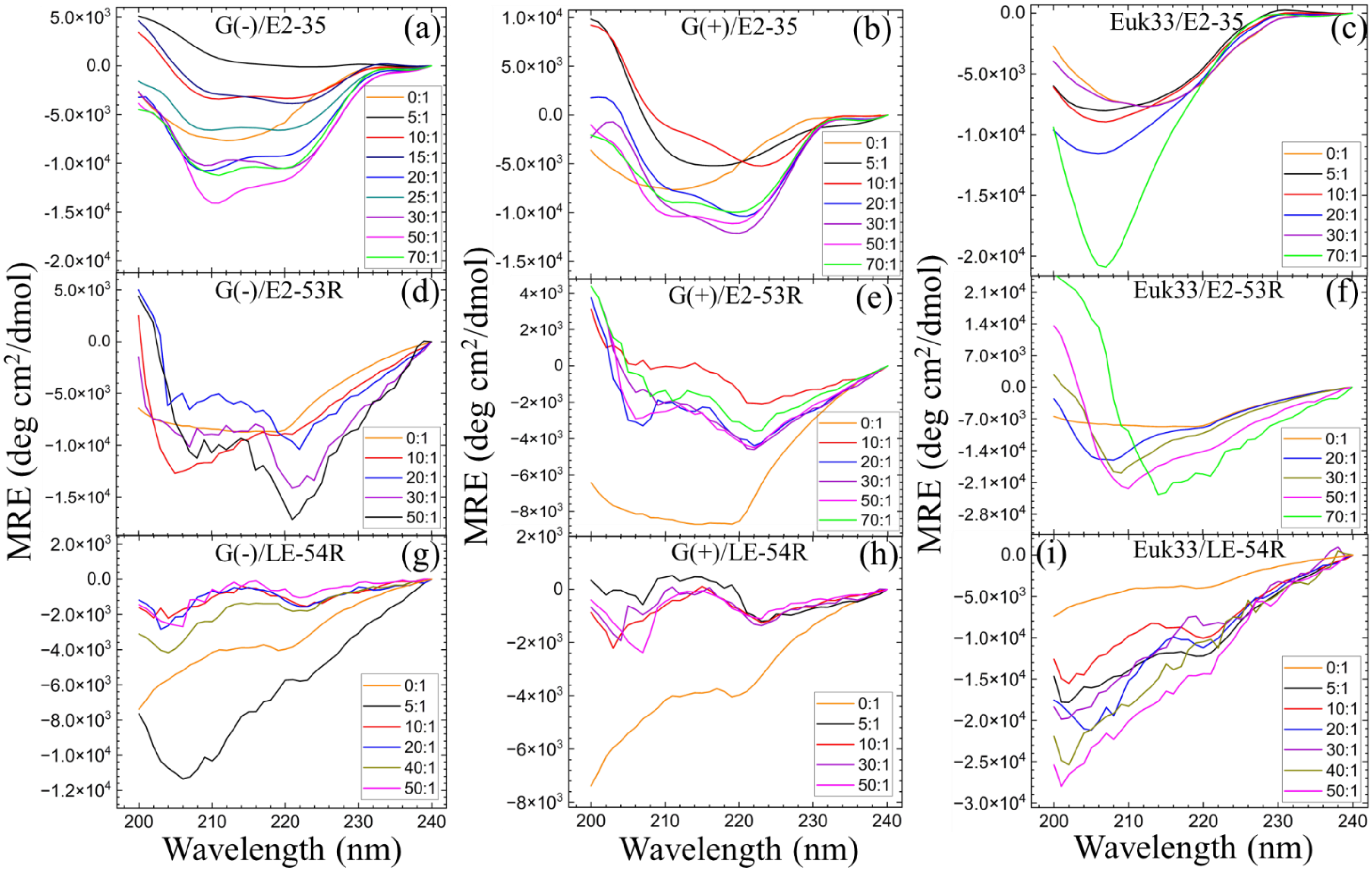

Figure S1. Mean residue ellipticity (MRE) results of E2-35 (**a, b and c**), E2-53R (**d, e and f**) and LE-54R (**g, h and i**) in G(-)IM, G(+) and Euk33 LMM ULVs. E2-35 data were adapted from our previously published paper [45] with permission.

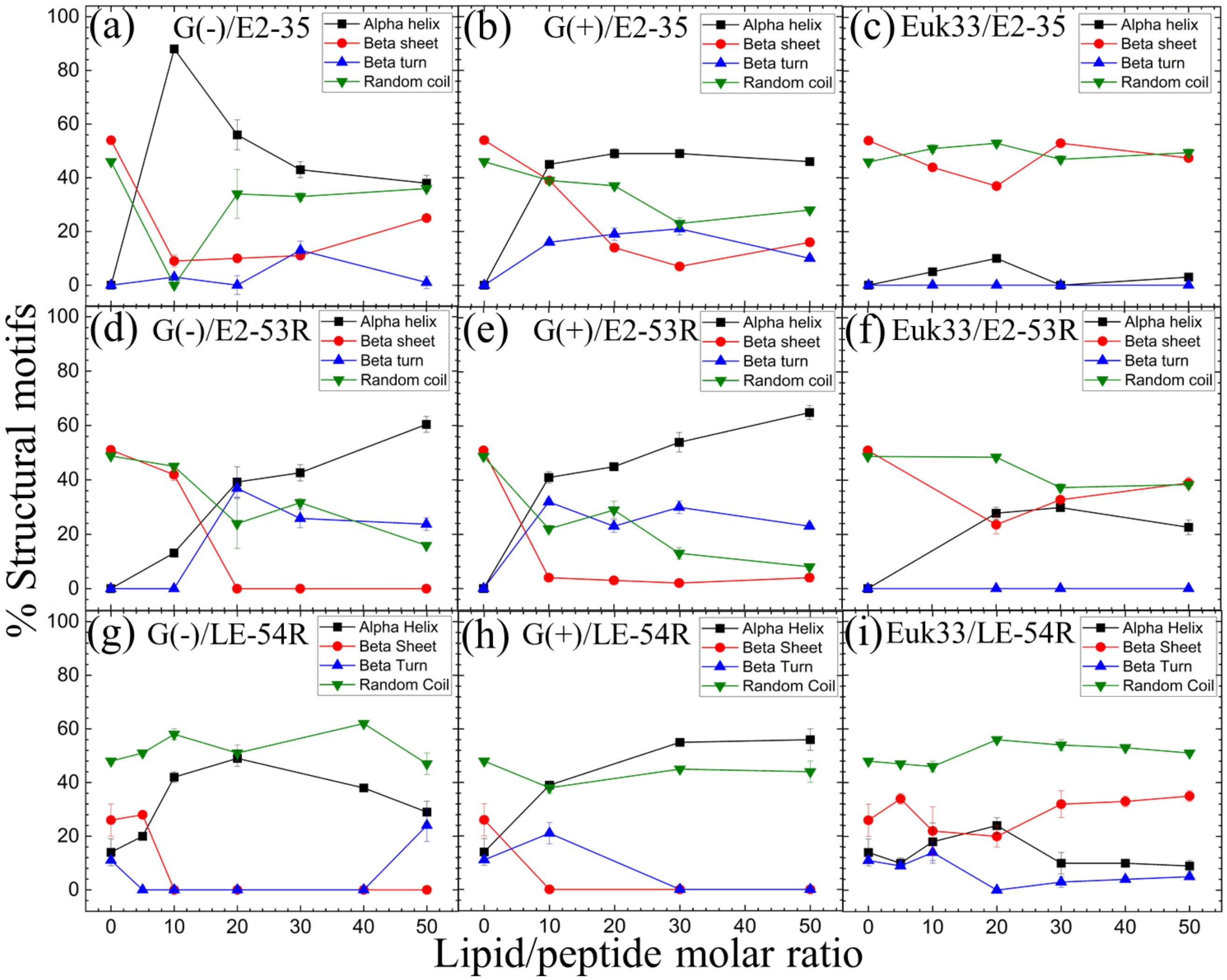

Figure S2. % Structural motifs vs. lipid/peptide molar ratio of E2-35 (**a, b and c**), E2-53R (**d, e and f**) and LE-54R (**g, h and i**) in three different LMM ULVs. Standard deviations represent 3-4 fitting results using shape analysis. E2-35 data were adapted from our previously published paper[45] with permission.

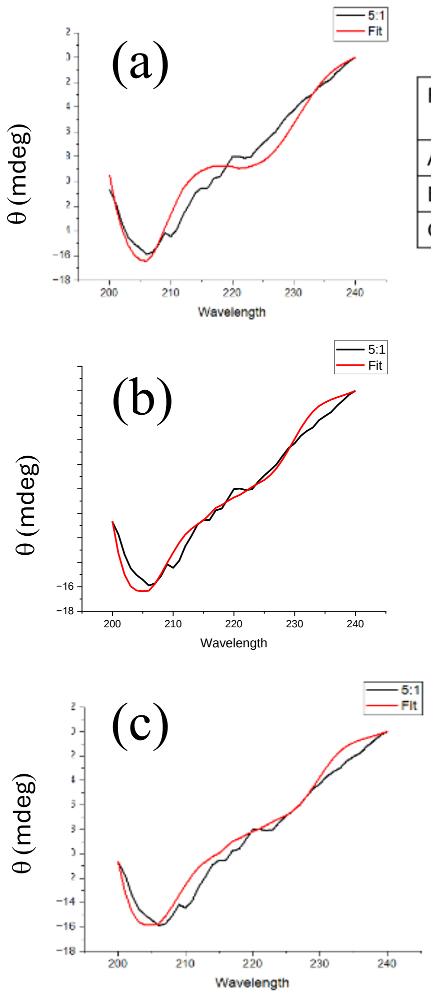

FIT	Alpha-helix(%)	Beta-sheet(%)	Beta-turn(%)	Random coil(%)	Adj. R^2
A	49	0	0	51	0.9472
B	20	29	0	51	0.9962
C	20	28	0	52	0.9949

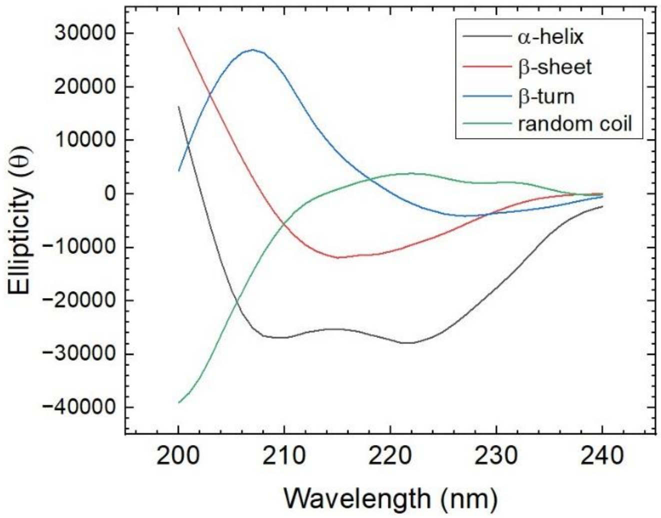

Figure S3. % Structural motifs (in table) for three different fits of the four secondary structural motifs shown above right from Ref. 67 to G(-)/LE-54R (5:1). (**a**) No weighting. (**b**) Weighted x10 at 200 and 240 nm. (**c**) Weighted x10 at 200, 220 and 240 nm. Levenberg/Marquardt fitting routine in Origin Pro 2024 was used to fit the data to the four literature secondary structural motifs. Averages and standard deviations were calculated from several fitting attempts for the same sample's ellipticity.

Tables S1 – S6 summarize secondary structural results (% α-helix, β-sheet, β-turn and random coil) for E2-53R and LE-54R peptides in three LMMs. R^2 indicates the goodness of fit.

Table S1. E2-53R CD results of secondary structure in G(-) IM LMMs

G(-) IM/E2-53R	α-helix (%)	β-sheet (%)	β-turn (%)	Random	Adj. R^2
0:1	0 ± 0	51.0 ± 0.4	0 ± 0	48.8 ± 0.3	0.98
10:1	13.1 ± 1.5	41.9 ± 2.2	0 ± 0	45.0 ± 1.2	0.99
20:1	39.2 ± 5.6	0 ± 0	36.9 ± 3.5	23.9 ± 9.1	0.97
30:1	42.6 ± 3.0	0 ± 0	25.8 ± 3.4	31.6 ± 1.5	0.93
50:1	60.4 ± 2.9	0 ± 0	23.7 ± 2.3	15.9 ± 0.7	0.97

Table S2. E2-53R CD results of secondary structure in G(+) LMMs

G(+) /E2-53R	α-helix (%)	β-sheet (%)	β-turn (%)	Random	Adj. R^2
0:1	0 ± 0	51.0 ± 0.4	0 ± 0	48.8 ± 0.3	0.98
10:1	41 ± 2.1	4 ± 0.1	32 ± 1.2	22 ± 1.3	0.92
20:1	45 ± 1.3	3 ± 1.3	23 ± 2.3	29 ± 3.2	0.92
30:1	54 ± 3.6	2 ± 0.8	30 ± 2.3	13 ± 2.1	0.87
50:1	65 ± 2.6	4 ± 1.6	23 ± 1.2	8 ± 1.1	0.84
70:1	51.3 ± 3.8	24.8 ± 2.6	24.1 ± 3.2	0 ± 0	0.97

Table S3. E2-53R CD results of secondary structure in Euk33 LMMs.

Euk33 /E2-53R	α-helix (%)	β-sheet (%)	β-turn (%)	Random	Adj. R^2
0:1	0 ± 0	51.0 ± 0.4	0 ± 0	48.8 ± 0.3	0.98
20:1	27.8 ± 2.2	23.6 ± 3.4	0 ± 0	48.5 ± 1.3	0.93
30:1	29.9 ± 1.4	32.8 ± 0.1	0 ± 0	37.3 ± 1.4	0.79
50:1	22.6 ± 2.7	39 ± 2.1	0 ± 0	38.4 ± 0.6	0.92
70:1	0	46 ± 0.7	19 ± 0.6	35 ± 0	0.94

Table S4. LE-54R CD results of secondary structure in G(-) IM LMMs.

G(-) IM/LE-54R	α-helix	β-sheet (%)	β-turn (%)	Random	Adj. R^2
0:1	14±5	26±6	11±1	48±1	0.97±0.01
5:1	20±1	28±1	0±0	51±1	0.99±0.01
10:1	42±2	0±0	0±0	58±2	0.93±0.07
20:1	49±3	0±0	0±0	51±3	0.90±0.03
40:1	38±1	0±0	0±0	62±1	0.98±0.02
50:1	29±4	0±0	24±6	47±4	0.90±0.03

Table S5. LE-54R CD results of secondary structure in G(+) LMMs

G(+)/LE-54R	α-helix	β-sheet (%)	β-turn (%)	Random	Adj. R^2
0:1	14±5	26±6	11±1	48±1	0.97±0.01
10:1	39±1	0±0	21±4	38±2	0.91±0.01
30:1	55±1	0±0	0±0	45±1	0.93±0.02
50:1	56±4	0±0	0±0	44±4	0.94±0.02

Table S6. LE-54R CD results of secondary structure in Euk33 LMMs

Euk33/LE-54R	α-helix	β-sheet (%)	β-turn (%)	Random	Adj. R^2
0:1	14±5	26±6	11±1	48±1	0.97±0.01
5:1	10±2	34±2	9±1	47±1	0.97±0.01
10:1	18±7	22±9	14±4	46±2	0.97±0.02
20:1	23±3	20±4	0±0	56±1	0.99±0.01
30:1	10±4	32±5	3±2	54±2	0.99±0.01
40:1	10±1	33±2	4±3	53±1	0.99±0.02
50:1	9±2	35±2	5±1	51±1	0.98±0.01

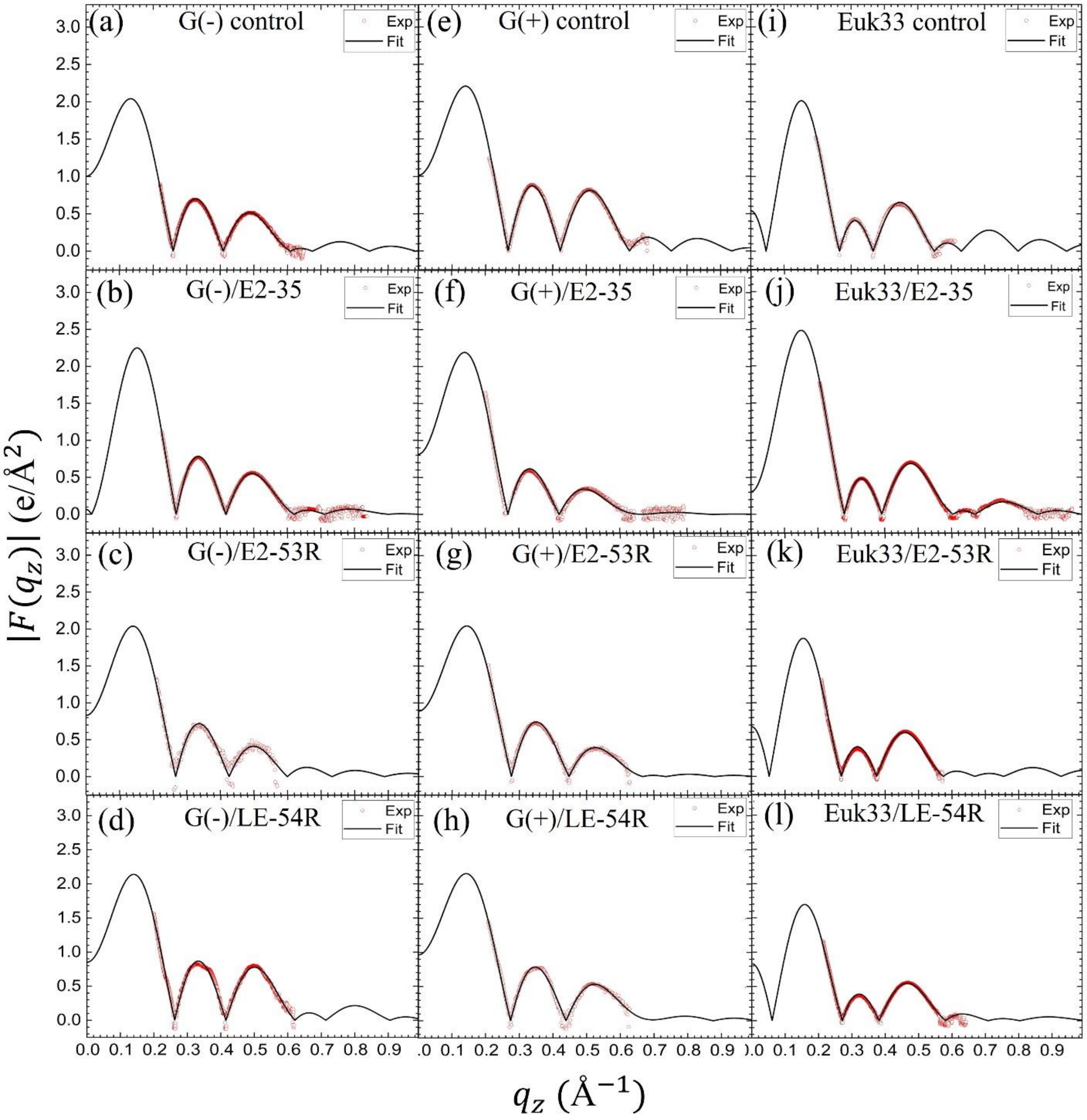

Figure S4. $|F(q_z)|$ for G(-) IM LMMs (**a – d**), G(+) LMMs (**e - h**) and Euk33 LMMs **(i - l)** in the presence of E2-35, E2-53R and LE-54R. The red points are experimental data, and the black line is the SDP model fit to the data. Lipid/peptide molar ratio is 50:1. E2-35 data are at lipid /peptide molar ratio of 75:1 and adapted from our published paper [45] with permission.

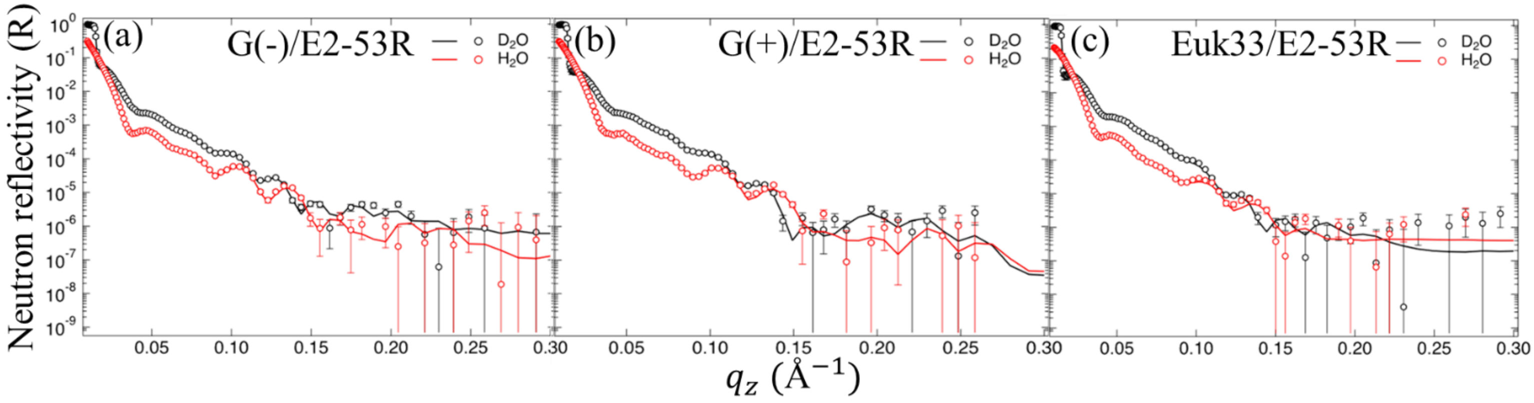

Figure S5. Neutron reflectivity (R) profiles of **(a)** G(-) IM LMMs, **(b)** G(+) and **(c)** Euk33 LMMs LMMs in the presence of E2-53R. The solid black and red lines are the fits to the D_2O and H_2O data respectively.

Table S7. NR results of E2-53R in G(-), G(+) and Euk33 LMMs

Parameter	G(-)/E2-53R	G(+)/E2-53R	Euk33/E2-53R
**Substrate**			
SiOx nSLD / 10^{-6} Å^{-2}	3.4± 0.1	3.8 ± 0.9	3.3 ± 0.2
SiOx thickness / Å	25.1 ± 9.6	25.4 ± 11.4	24.8 ± 14.7
Cr nSLD / 10^{-6} Å^{-2}	3.3± 0.3	3.3 ± 0.5	3.3 ± 0.45
Cr thickness / Å	23.9 ± 9.4	26.5 ± 12.3	24.2 ±9.4
Au nSLD / 10^{-6} Å^{-2}	4.5 ± 0.1	4.5 ± 0.1	4.5 ± 0.13
Au thickness / Å	152.9 ± 3.3	149.5± 0.9	152 ± 5.6
**Bilayer**			
R.M.S. roughness / Å	3 ± 1	5 ± 1	6 ± 2
Tether thickness / Å	10 ± 1	9 ± 1	12 ± 2
Hydrocarbon thickness inner lipid leaflet / Å	15 ± 3	17 ± 3	17 ± 3
Hydrocarbon thickness outer lipid leaflet / Å	19 ± 3	15 ± 3	15 ± 4
Area per lipid, outer leaflet / Å^2	60 ± 10	70 ± 10	60 ± 20
Bilayer completeness / %	95 ± 3	98 ± 2	91 ± 8
**Peptide**			
Amount of membrane-associated protein / Å^3/Å^2	9 ± 2	2 ± 1	6 ± 2
Fraction of protein in hydrocarbons	0.24±0.08	0.41±0.15	0.67±0.17
Fraction of protein in headgroups	0.14±0.05	0.25±0.08	0.24±0.12
Fraction of protein in bulk solvent	0.61±0.10	0.27±0.18	0.02±0.02
Peak position from headgroup / solvent interface / Å	-10 ± 10	n/a (too little protein)	-30 ± 15 (wide plateau)
**General**			
Goodness of fit, chi-squared	2.3	3.1	3.6
